# Prevalence of emotional blunting and its association with childhood adverse experiences in depressive episodes

**DOI:** 10.1186/s12888-026-08518-8

**Published:** 2026-08-13

**Authors:** Narak Chadbunchachai, Papan Vadhanavikkit, Sirinapa Aphisitphinyo, Pattharee Paholpak, Nawanant Piyavhatkul, Parinda Pienroj, Pongsatorn Paholpak

**Affiliations:** Department of Psychiatry, Faculty of Medicine, Khon Kaen University, Khon Kaen, 40000 Thailand

**Keywords:** Emotional blunting, Depressive episode, Adverse childhood experiences, Prevalence

## Abstract

**Background:**

Emotional blunting (EB) is common in patients with depressive episodes. This study aimed to study its prevalence in remitted and mild-to-moderate depressive episodes, and explore its association with adverse childhood experiences (ACEs).

**Methods:**

This cross-sectional study included patients aged 18–65 years with mild depressive episodes in major depressive disorder (MDD), persistent depressive disorder (PDD), and bipolar disorder (BPD). EB and adverse childhood experiences were assessed using the Oxford Depression Questionnaire (ODQ) and the ACE questionnaire, respectively. The severity of depressive symptoms was measured using the Hamilton Depression Rating Scale (HAM-D). Statistical analyses used Pearson’s correlation coefficients.

**Results:**

A total of 106 participants completed the study. Prevalence of EB based on a single-question screening and ODQ cut-off score of ≥ 26 were 55.6%, and 89%, respectively. There were no significant differences in prevalence among diagnostic groups (*p* > 0.05). The prevalence was significantly lower in remitters (HAM-D ≤ 7) than non-remitters (45.3% vs. 66%, X^2^= 4.97, *p* < 0.05). Correlation analyses in entire sample showed significant correlations between the ODQ total score and HAM-D score, PHQ-9 score, and the number of depressive episodes (all *p* < 0.05). The Not Caring (NC) domain of the ODQ was positively correlated with ACE subscale scores for emotional abuse (*r* = 0.259, *p* = 0.007) and physical abuse (*r* = 0.208, *p* = 0.033), and negatively correlated with emotional support (*r* = − 0.25, *p* = 0.01). Subgroup analyses among remitters (*n* = 53) showed that NC scores were correlated with emotional abuse (*r* = 0.304, *p* = 0.027) and emotional support (*r* = − 0.328, *p* = 0.017). The emotional detachment (ED) domain of the ODQ was correlated with the ACE domestic violence subscale (*r* = 0.286, *p* = 0.038).

**Conclusion:**

EB was present in more than half of patients with depressive episodes. Remission was associated with a lower prevalence of EB; however, the prevalence remained high even among remitters. The severity of EB was correlated with the severity and recurrence of depressive symptoms. The Not Caring domain was associated with reported emotional abuse and domestic violence during childhood, even among remitted patients.

## Introduction

Major depression or major depressive disorder is one of the most common mental health disorders globally. It is estimated that approximately 332 million people worldwide currently live with depression, with roughly 5.7% of adults globally suffer from depression [1]. The pandemic of COVID-19 triggered a massive spike in global mental health struggles. Epidemiological models indicate that the global prevalence of Major Depressive Disorder (MDD) increased by roughly 27% in 2020 alone due to the stress, isolation, and socioeconomic impacts of the pandemic, and incidence rates have remained historically high in the years following [2]. While MDD can occur at any stage of life, incidence rates typically surge during adolescence and young adulthood (ages 15–29). MDD is a leading cause of Years Lived with Disability (YLDs) globally. Recent GBD data indicates that depressive disorders account for over 56 million Disability-Adjusted Life Years (DALYs) [3] worldwide [4]. Nearly 90% of individuals diagnosed with depression report significant difficulty managing work, home, or social activities due to their symptoms [5]. Depression is not a disorder with a single cause; rather, it stems from multifactorial influences, including neurobiological changes, genetic predisposition, physical health conditions, behavioral risks, and adverse life experiences. Childhood adverse experiences, including but not only abuse, neglect, bullying, and domestic violence are among the strongest predictive environmental risk factors for developing MDD [6], and associated with poor response and frequent recurrence [7, 8]. According to a meta-analysis, childhood trauma is common among patients with major depression, with a prevalence rate of up to 62% [9].

Emotional blunting (EB), characterized by a reduction in emotional responsiveness, is a significant phenomenon observed across various neurological and psychiatric conditions, particularly MDD [10]. EB is an umbrella term which covers apathy, amotivation, anhedonia, emotional numbness, and lack of empathy. Many patients with EB reported either childhood or recent psychological trauma [11]. Prevalence of EB in patients with MDD differs along the course of illness. During the acute phase of depression EB might be found up to 75–91.9%, while the prevalence declines to 25–73.3% after the treatment [10, 12]. 

The underlying neuropathology of emotional blunting (EB) is not yet well understood, but it is hypothesized to involve decreased dopaminergic or glutamatergic activity in the prefrontal cortex. This hypothesis aligns with the well-documented adverse effects of selective serotonin reuptake inhibitors (SSRIs), which include emotional indifference and an inability to cry [13, 14]. These experiences cause negative effects to overall well-being of the patients. A recent large web-based survey study from Japan demonstrated moderate correlation between EB and functional impairment [15], while another recent study from India found that up to 40% of patients considered stopping the antidepressant due to emotional blunting [16], which might led to medication discontinuation in up to one-third of patients with MDD [13, 14, 17]. Despite these clinical impacts, gaps in our knowledge regarding EB in major depression remain, particularly concerning patients in remission or those with mild-to-moderate depression and a history of childhood trauma.

This study was conducted to explore the prevalence of EB in patients with remitted or mild to moderate depression, and to examine the association between EB and childhood trauma. We hypothesized that the prevalence of EB in patients with remitted and mild-to-moderate depression would be approximately 40–50%. Additionally, we postulated that the intensity of EB in remitted patients is significantly associated with childhood adverse experiences.

The sample size was calculated a priori based on the prevalence of EB of 46% among patients with MDD receiving treatment [13] with an estimated absolute error of 10%, and a power of 80%. Assuming a 10% rate of incomplete data, the required sample size was calculated to be 106 [18]. In addition, the expected correlation between EB and childhood adverse events was low (*r* = 0.3), therefore the sample size to test the second hypothesis was 85 [19]. Accordingly, the study set a sample size of 106 to adequately address both hypotheses.

### Ethic approval and consent to participate

This study was conducted in accordance with the ethical principles of the Declaration of Helsinki and relevant institutional guidelines for research involving human participants. The study protocol was reviewed and approved by the Institutional Review Board/Ethics Committee of Center for Ethics in Human Research, Khon Kaen University under approval number #HE661181.

All participants were informed about the objectives, procedures, potential risks, and benefits of the study before enrollment. Participation was entirely voluntary, and written informed consent was obtained from each participant prior to data collection. For older participants who required assistance in reading or understanding the study information, the details were explained clearly by the research team, and adequate time was provided for questions before consent was obtained. Participants were assured that refusal to participate or withdrawal from study at any time would not affect their medical care or access to treatment. Personal identifying information was removed from the research database, and all data were coded and stored securely with access limited to authorized members of the research team only.

## Methods

This cross-sectional study was conducted at a medical university hospital in Thailand. The study was carried out over 12 months, from October 2023 to November 2024. The study included patients aged 18–65 who were diagnosed with a mild to moderate depressive episode, defined by a Patient Health Questionnaire (PHQ-9) score of ≤ 18 during screening from routine outpatient visit. Participants had a pre-existing diagnosis of major depressive disorder (MDD), Bipolar I disorder (BPID), or persistent depressive disorder (PDD) based on DSM-5 criteria. After being assessed by psychiatrists for routine care using the Hamilton Depression Rating Scale (HAM-D), if the severity of symptoms was not severe, and non-suicidal then participants provided written informed consent. All patients had been on the same antidepressant regimen for at least one month. Individuals were excluded if their medical records indicated an intellectual disability or any pre-existing neurodevelopmental disorder.

Because the study involved assessment of depressive symptoms, participants who were found to have clinically significant depressive symptoms or strong suicidal idea/intention were advised to seek evaluation and appropriate management. When necessary, referral to a psychiatrist or mental health professional was arranged according to the clinical practice of the study setting. No experimental intervention was performed, and the study procedures were considered to involve minimal risk to participants.

After obtaining written informed consent, sociodemographic and clinical data were collected via interviews conducted by trained research assistants. Screening for emotional blunting was performed using a single question from the Oxford Depression Questionnaire (ODQ) probing for the emotional effects of antidepressants: “Emotional effects vary, but may include, for example, feeling emotionally ‘numbed’ or ‘blunted’ in some way; lacking positive emotions or negative emotions; feeling detached from the world around you; or ‘just not caring’ about things that you used to care about. Have you experienced such emotional effects during the last six weeks?” Following this probing question, all participants self-completed the ODQ and the Adverse Childhood Experiences (ACE) Questionnaire.

### Measurements

#### Oxford depression questionnaire (ODQ)

The ODQ is a 26-item self-report scale specifically designed to measure emotional blunting—the feeling of being emotionally “numb” or “dulled.” The ODQ is divided into four main dimensions: General Reduction (GR), Reduction in Positive Emotion (RP), Emotional Detachment (ED), and Not Caring (NC). An optional fifth dimension assesses Antidepressant as Cause (AC). It is scored by summing responses on a 5-point Likert scale, where each item is rated from 1 to 5 based on the level of agreement: 1 = Disagree; 2 = Disagree a little; 3 = Neither agree nor disagree; 4 = Agree a little; and 5 = Agree. Higher scores indicate a greater intensity of emotional blunting, categorized as follows: 26–52 (minimal), 53–78 (mild), 79–104 (moderate), and 105–130 (severe) [20]. The author obtained permission from Oxford University Innovation Limited to translate the ODQ into the Thai language, strictly following guidelines for cultural validation. The Thai versions of the ODQ-26 and ODQ-20 demonstrated Cronbach’s alpha values of 0.886 and 0.868, respectively, within the population of this study.

#### Adverse childhood experiences (ACE)

The Adverse Childhood Experiences (ACE) questionnaire is a 26-item self-report tool used to assess major types of childhood trauma, including emotional abuse, sexual abuse, physical neglect, emotional neglect, and domestic violence. This study utilized the original CDC-Kaiser ACE questionnaire, which quantifies the frequency of each trauma type. Most items use a 5-point Likert scale ranging from 0 (never) to 4 (very frequent), where higher scores indicate a greater frequency of abuse or neglect. However, four items related to sexual abuse and six items related to household dysfunction use a dichotomous rating (0 = no, 1 = yes) [21]. Additionally, five items assessing emotional neglect use reverse scoring; for these items, a higher sum score indicates frequent emotional support from the family, hereafter referred to as “emotional support”. The Thai version of the original CDC-Kaiser ACE questionnaire has been culturally validated with a Cronbach’s Alpha of 0.856 [22]. 

#### Hamilton depression rating scale (HAMD-17)

The Hamilton Depression Rating Scale (HDRS or HAMD) is a clinician-administered psychometric questionnaire. This study utilized the 17-item version (HAMD-17), which employs two distinct scoring systems. A 5-point Likert scale (0–4) ranging from “absent” (0) to “very severe” [4] for symptoms that allow for more nuanced measurement, such as depressed mood, guilt, and impaired work and activities. A 3-point Likert scale (0–2): Ranging from “absent” (0) to “present” [2] for symptoms that are more difficult to quantify, such as weight loss, insomnia, and gastrointestinal symptoms. The total score is categorized as follows: 0–7 (remission), 8–13 (mild), 14–18 (moderate), and 19 or higher (severe). The Thai version of HAMD was validated previously with Cronbach’s alpha value of 0.858 [23].

#### The patient health questionnaire-9 (PHQ-9)

The Patient Health Questionnaire-9 (PHQ-9) is one of the most common and widely used self-report tools for screening, diagnosing, and measuring the severity of depression. It asks the patient to rate how often they have been bothered by 9 specific symptoms over the past two weeks. Each of the 9 items is scored from 0 to 3 based on frequency: 0 = Not at all; 1 = Several days; 2 = More than half the days; 3 = Nearly every day. The total score is a sum from every item; the higher score indicates greater severity of depressive symptoms. This study used PHQ-9 as a screening tool to capture patients with mild – moderate depression, which the total score falls within the range of 0–18. The Thai version of PHQ-9 was validated previously with Cronbach’s alpha value of 0.79 [24]. 

### Statistical analysis

Data analyses used SPSS version 28. Descriptive statistics used percentages for categorical variables and means ± standard deviations (SD). Analytical statistics for differences between two groups used the independent t-test, and the Pearson correlation coefficient for correlation analyses. Statistical significance was set at a p-value less than 0.05.

## Results

A total of 106 participants were recruited and completed the study with demographic data detailed in the Table 1. Prevalence of EB from the single-question screening and a cut-off of ODQ ≥ 26 were 55.6%, and 89%, respectively. Using the single-question screening, the prevalence of EB in MDD, PDD, and BPD were 50.8%, 63.2%, and 61.5%, respectively. The prevalence was not associated with diagnostic groups (*p* > 0.05). Among remitters (*n* = 53; 50%), whose HAM-D score ≤ 7, the prevalence of EB was significantly lower than non-remitters (45.3% vs. 66%, chi’s square = 4.97, *p* < 0.05). Regarding ODQ score 67 participants (63.2%) had minimal EB, 38 had mild EB (35.8%), and 1 had severe EB (0.9%). For the total ODQ scores, both ODQ-26 and ODQ-20, non-remitters had significantly higher scores than remitters (both *p* < 0.05). There were 2 domains, not care (NC), and reduction in positive emotion (RP), which scores were significantly higher in non-remitters (both *p* < 0.05). All patients were on at least one antidepressant, and 97 (91.5%) participants reported score from antidepressant as cause (AC) domain greater than zero. Mean of the ACE total scores were not significantly different between remitters and non-remitters (X_remitters_ = 17.5 ± 6.5 vs. X_non−remitters_ 19.4 ± 6.5, df = 104, *p* = 0.131). The means from all ACE subscale scores, were not different between remitters and non-remitters (all *p* > 0.05).

**Table 1:** Demographic data of the participants

Demographic (*N* = 106)	Mean ± SD, *N* (%)
Age (years)	29 ± 9.97
Sex (female)	77 (72.6%)
Education (years)	15.0 ± 2.0
Employment	67 (63%)
Monthly income (USD)	738 ± 1,833
Diagnoses	
-Major Depressive Disorder -Persistent Depressive Disorder -Bipolar Disorder	61 (57.5%) 19 (17.9%) 26 (24.6%)
Duration of illness (years)	2.2 ± 0.8
Number of total mood episodes	4 ± 3.3
HAM-D -remission (0–7) -mild depression (8–16) -moderate depression (17–23)	8.1 ± 5.9 53 (50%) 40 (37.7%) 13 (12.3%)
ODQ -not caring -general reduction -emotional detachment -reduction of positive emotion -antidepressant as cause	46 ± 17.1 9 ± 4.7 11.2 ± 3.5 7.5 ± 5.2 10.9 ± 5.2 7.8 ± 5.3
ACE	
emotional abuse -physical abuse -domestic violence -physical neglect -sexual abuse -sum of abuse + violence + neglect -emotional support	3.2 ± 2.3 1.6 ± 1.8 1.3 ± 2.9 3.9 ± 3.2 0.5 ± 1.1 10.5 ± 7.5 12.3 ± 5.0

HAM-D = Hamilton depressive symptoms rating scale; ODQ = Oxford Depression Questionnaire; ACE = Adverse Childhood Experiences

**Table 2:** Pearson’s correlation coefficients between the subscale scoresfrom oxford depression questionnaire (ODQ) and adverse childhood experiences (ACE)

*N* = 106	Adverse Childhood Events					
	Emotional abuse	Physical abuse	Sexual abuse	Emotional Support	Domestic Violence	Physical Neglect
Oxford Depression Questionnaire						
Not caring	0.259**	0.208*	0.023	-0.25**	0.05	0.103
General reduction	0.129	0.039	0.023	-0.110	0.179	0.199*
Emotional detachment	0.132	0.110	-0.11	-0.118	0.16	0.09
Reduction of positive emotion	-0.033	0.044	-0.35	-0.114	0.004	-0.006
Antidepressant as cause	0.185	0.066	0.041	-0.020	0.059	0.109
Total score	0.184	0.125	-0.016	-0.168	0.123	0.132

*P < 0.05; ** *p* < 0.01

Correlation analysis using Pearson’s correlation coefficients between ODQ and demographic variables showed that there were significant correlations between the total ODQ score and following demographic and clinical variables: HAM-D score (*r* = 0.232, *p* = 0.016), PHQ-9 score (*r* = 0.36, *p* < 0.001), and number of recurrent episodes (*r* = 0.268, *p* = 0.035). The HAM-D score was significantly correlated with both the NC domain (*r* = 0.287, *p* = 0.003), and the RP domain (*r* = 0.378, *p* < 0.001) scores of the ODQ. Similarly, the PHQ-9 score was significantly correlated with the NC domain (*r* = 0.409, *p* < 0.001), and the RP domain (*r* = 0.568, *p* < 0.001) scores of the ODQ. Only the PHQ-9 score had a statistically significant correlation with physical abuse subscale score (*r* = 0.254, *p* = 0.09).

Analysis of the total population found that the NC domain score of the ODQ was significantly correlated with the following ACE subscale scores: emotional abuse (*r* = 0.259, *p* = 0.007); physical abuse (*r* = 0.208, *p* = 0.033); and emotional support (*r* = -0.25, *p* = 0.01). Additionally, the General Reduction (GR) domain score was significantly correlated with the ACE physical neglect score (*r* = 0.199, *p* = 0.041), and the RP domain score of the ODQ was significantly correlated with the number of recurrent episodes (*r* = 0.347, *p* = 0.006). After controlling the AC domain score using partial correlation analysis, the only statistically significant correlation was the negative correlation between the NC domain score of the ODQ and the ACE emotional support score (*r* = -0.233, *p* = 0.018). All correlation coefficients were detailed in Table 2.

Among remitters, there were no significant correlations between the total ODQ score and either HAM-D, or PHQ-9 scores. Neither the PHQ-9 nor the HAM-D scores were correlated with any ACE subscale score. However, the NC score was significantly correlated with the following ACE scores: emotional abuse (*r* = 0.304, *p* = 0.027), and emotional support (*r* = -0.328, *p* = 0.017). Furthermore, emotional detachment (ED) score was significantly correlated with the ACE domestic violence score (*r* = 0.286, *p* = 0.038).

## Discussion

To the best of our knowledge, this study was the first study which explores the prevalence of emotional blunting in patients with mild to moderate major depressive episode from MDD, PDD and Bipolar I disorder and its associated factors. Emotional blunting was found in more than half of the patients, regardless of the diagnosis. This study found that the intensity of this emotional phenomenon was correlated with severity of adverse childhood experiences while it was not correlated with the severity of depressive symptoms in the patients with remitted depression. We discussed our findings and compared them with literature below.

Emotional blunting was highly prevalent in this study, with rates consistent with previous research using the ODQ ranging from 25% to 91.0% [12, 15, 16, 20]. These variations in prevalence across studies can be attributed to several factors, including measurement differences, the specific characteristics of mood disorders, and cultural nuances. First, there are no universally accepted diagnostic criteria or standardized measurements for emotional blunting. Even when using the ODQ, utilizing a single screening question yields different prevalence rates compared to using established cut-off scores [20]. Second, the variety of tools used across literature likely explains these discrepancies. The ODQ was the first scale developed specifically for this purpose; however, it captures prevalence through a single item, while 26 items are used to assess severity and subdomains. Other scales, such as the Snaith–Hamilton Pleasure Scale (SHAPS) or the anhedonia subscales of the HAM-D and Montgomery-Asberg Depression Rating Scale (MADRS), may capture elements of emotional blunting, particularly anhedonia, but they lack the detail required to assess domains like emotional numbness and detachment from ODQ. Both the PHQ-9 and the HAM-D which this study used also were correlated with certain ODQ domains, particularly the NC and the RP. These findings suggest that traditional measurements for depressive disorders could partially capture EB. Third, cultural differences in the regions where these studies were conducted may influence reported prevalence. It is well known that the prevalence of major depression varies globally. For instance, many East Asian countries, such as Japan and Korea, report lower rates of depressive disorders compared to Western nations, yet they have significantly higher suicide rates. This discrepancy strongly suggests that coping styles and the clinical presentation of symptoms differ significantly across cultures.

Regarding population characteristics, most previous studies recruited only patients with MDD, excluding those with Bipolar I disorder (experiencing a depressive episode) or Persistent Depressive Disorder (PDD). This study included all three diagnostic groups and found no significant differences in prevalence between them. This finding contributes a vital piece of information to literature; emotional blunting appears to be a phenomenon linked to the polarity of the mood state rather than a specific diagnosis. While previous research found that emotional blunting occurs in both exacerbated and stabilized depressive episodes [10], our study focused on patients whose medication had been stable for at least one month. We found a significantly lower prevalence of emotional blunting in remitters compared to non-remitters. Furthermore, the severity of emotional blunting correlated with depressive symptom scores and the number of lifetime depressive episodes. These findings suggest that both the chronicity and severity of mood disorder influence the intensity of emotional blunting, even in a non-severely depressed population. In this context, emotional blunting likely represents a residual mood symptom rather than an acute manifestation of mood disturbance. However, since most participants were taking at least one antidepressant, we cannot rule out the possibility that the blunting was a side effect of the treatment itself. Nearly all participants subjectively attributed their emotional blunting to their medication—this is supported by previous studies associating the condition with SSRIs, SNRIs, and combination treatments involving antipsychotics [13, 14, 25]. 

The correlations found between frequency of ACE and EB in this study are noteworthy. All participants reported at least one ACEs, particularly a lack of emotional support, emotional abuse, and physical neglect. However, the frequency of ACEs, and emotional support were not different between remitters and non-remitters. The significant positive correlations between the NC subscale and both emotional and physical abuse, alongside the significant negative correlation with emotional support, indicate that frequent childhood traumas impact the “intensity of caring” in depressed patients. In addition, the positive correlation between the PHQ-9 and the physical abuse subscale suggests that the severity of depression was linked to the frequency of childhood physical abuse. Crucially, the correlations between emotional abuse, emotional support, and the NC domain remained significant even among remitters. These findings strongly suggest that emotional abuse and a lack of emotional support negatively impact attachment and decrease the ability to care about one’s surroundings. This is well-supported by existing preclinical and clinical data from both rodents and humans, that early adverse experiences disturb reward processes specifically affecting reward gauging, perception, and decision-making [26]. Furthermore, the significant positive correlation between the ED domain and the frequency of domestic violence among remitters suggests that childhood domestic violence can cause serious impairment to attachment. This may lead patients to feel “detached” from people and their environment, even when they are no longer symptomatically depressed. It is worth noting that no significant correlation was found between sexual abuse and any ODQ domain. This is likely due to the relatively low prevalence (25%) of reported sexual abuse in this study, whereas other ACE domains were reported in more than 60% of participants. Furthermore, the negative correlation between the NC domain and emotional support, after controlling for the AC domain score, strongly suggests that NC intensity may be independently driven by childhood trauma specifically, a lack of emotional support.

Taken altogether, childhood traumas have been consistently linked to mood disorders, particularly treatment-resistant depression [8]. ACEs negatively impact the developing brain especially the reward circuit. Given that the construct of emotional blunting relies heavily on the perception of pleasure, connection, and motivation, trauma-induced impairment of reward perception likely contributes to the diminished capacity for these emotional experiences.

## Conclusion

In summary, this study found that emotional blunting is prevalent across remitted mood disorders, regardless of the operational definition used, and its severity correlates with a history of childhood adverse events. However, major limitations include the single-site cohort design, a relatively small sample size, the lack of quantified anxiety symptoms, and the absence of a remitted control group not taking antidepressants. Furthermore, as with other retrospective studies on childhood adversity, recall bias was unavoidable. Future research involving larger, multi-site populations and more rigorous methodologies, including potential treatment interventions, is needed to better understand this complex psychological symptom and its clinical implications.

## Data Availability

The datasets generated and/or analyzed during the current study are not publicly available due to privacy and ethical restrictions but are available from the corresponding author on reasonable request.

## Abbreviations

MDD: Major depressive disorder
YLDs: Years Lived with Disability
DALYs: Disability-Adjusted Life Years
EB: Emotional blunting
SSRIs: Selective serotonin reuptake inhibitors
PHQ-9: Patient Health Questionnaire
BPID: Bipolar I disorder
PDD: Persistent depressive disorder
HAM-D: Hamilton Depression Rating Scale
ODQ: Oxford Depression Questionnaire
ACE: Adverse Childhood Questionnaire
GR: General Reduction
RP: Reduction in Positive Emotion
ED: Emotional Detachment
NC: Not Caring
AC: Antidepressant as Cause
